# Postural stability and visual impairment: Assessing balance in children with strabismus and amblyopia

**DOI:** 10.1371/journal.pone.0205857

**Published:** 2018-10-18

**Authors:** Anat Bachar Zipori, Linda Colpa, Agnes M. F. Wong, Sharon L. Cushing, Karen A. Gordon

**Affiliations:** 1 Department of Ophthalmology and Vision Sciences, The Hospital for Sick Children, Toronto, Canada; 2 Department of Ophthalmology, Tel Aviv Sourasky Medical Center, affiliated to the Sackler Faculty of Medicine, Tel Aviv University, Tel Aviv, Israel; 3 Eye Movement and Vision Neuroscience Laboratory, The Hospital for Sick Children, Toronto, Canada; 4 Department of Otolaryngology–Head and Neck Surgery, The Hospital for Sick Children, Toronto, Canada; 5 Archie's Cochlear Implant Laboratory, Cochlear Implant Program and Communication Disorders, The Hospital for Sick Children, Toronto, Canada; Moorfields Eye Hospital NHS Foundation Trust, UNITED KINGDOM

## Abstract

**Background:**

Vision plays an important role in controlling posture and balance in children. Reduced postural control has been reported in children with strabismus, but little has been reported specifically in amblyopia.

**Objective:**

To investigate whether children with amblyopia have reduced balance compared to both children with strabismus without amblyopia and healthy controls.

**Study design and methods:**

In this cross-sectional study, a total of 56 patients and healthy controls were recruited from the Ophthalmology and Otolaryngology Clinics at The Hospital for Sick Children, Toronto. Participants were divided into three groups: (1) 18 with unilateral amblyopia (strabismic amblyopia or mixed mechanism); (2) 16 with strabismus only without amblyopia; and (3) 22 visually-normal controls. The primary outcome was the balance performance as measured by the balance subtest of the Bruininks-Oseretsky Test of Motor Proficiency 2 [BOT2].

**Results:**

The age and gender-adjusted BOT2 balance scores were significantly reduced in the amblyopia group (mean score 9.0 ± 3.1 SD) and the strabismus without amblyopia group (mean score 8.6 ± 2.4 SD) compared to visually normal controls (mean score 18.9 ± 4.2) (p<0.0001), but no statistical difference was demonstrated between the two patient groups (p = 0.907). Further subgroup analysis of the strabismus only group did not reveal a statistically significant difference in performance on BOT2 balance score between strabismus only patients with good stereopsis 60 sec or better (BOT2 mean score 9.8±3.0 SD) to patients with 3000 sec or no stereopsis (BOT2 mean score 7.9±1.7) (p = 0.144).

**Conclusion:**

Our findings suggest that normal vision plays an important role in the development and maintenance of balance control. When normal binocular vision is disrupted in childhood in strabismus and/or amblyopia, not only is the vision affected, but balance is also reduced. Our results indicate that the presence of even mild binocular discordance/dysfunction (patients with intermittent strabismus and good stereopsis) may lead to postural instability.

## Introduction

Postural stability (balance) is essential for the achievement of developmental milestones [1]. It requires mastery of both static (sitting and standing) and dynamic (walking and running) postural control [2, 3], both of which are fundamental to the normal development of gross and fine motor skills [4]. Motor development is essential for the acquisition of a variety of skills in a timely fashion [1, 5]. Abnormalities in balance and coordination in school-aged children may lead to impaired academic performance and delayed social development, and may also affect the child’s general well-being and self-esteem [6]. It may also compromise the child’s safety. Balance improves with age and occurs more rapidly in girls than boys until they reach their teens; afterwards, males seem to have slightly better postural stability [7, 8].

Balance is dependent on the integration of inputs from a triad of sensory systems: visual, somatosensory (mainly proprioception) and vestibular [9–12]. With increasing age, there is a progressive domination of the somatosensory and vestibular systems, with less reliance on visual orientation inputs [7, 8, 13, 14]. Hence, it is reasonable to postulate that the proper development of the visual system is crucial to the development of normal balance. Equal and optimal visual acuity, binocular vision (stereopsis), and vergence eye movements should all contribute to postural stability. Unfortunately, the contributions of the various elements of vision to balance control are not fully confirmed [15–20]. There is conflicting evidence regarding the effect of monocular viewing on postural control [15, 17, 19]. Previous reports showed that balance could be affected by mild visual disorders [21, 22]. In one report, as many as 5% of children who presented with vertigo or disequilibrium had normal vestibular and somatosensory neurologic examinations, but instead were found to have visual disorders, such as vergence dysfunction, latent strabismus with binocular vision abnormalities, or anisometropia [21]. Most of these children did not have true vertigo, suggesting that their vision disorder was the source of the difficulties maintaining equilibrium. When the postural stability of these children was tested, they performed worse than their normal peers when fixating on a target at both near and far distance, and with binocular viewing, monocular viewing, or with both eyes closed [22].

Impaired balance and motor function has been reported in children with strabismus [16, 23–26]. Deficiencies were described in both static postural control (postural sway as measured on a posturography platform) [16, 23–27] and dynamic postural control (gait parameters) [23, 28]. It has also been previously reported that correcting their strabismus can improve motor development [25, 27, 29, 30], but not in the immediate postoperative period [31]. However, these reports included a heterogeneous group of patients (including esotropic, exotropic and vertical forms of strabismus in one group). Of note is that all of the reported patients had good and equal vision in both eyes [16, 25, 26].

Because postural control is reduced in children with strabismus, we hypothesize that it is further reduced in children with amblyopia. Amblyopia is a developmental neural abnormality that results in reduced visual acuity, typically monocular, that cannot be fully explained by a structural ocular disorder. It occurs during development when binocular images are either blurry or misaligned; the most common causes for amblyopia are ocular misalignment (strabismus) or a refractive error (anisometropia) or both (mixed mechanism) [32]. The mismatched input from each eye during development also leads to abnormal binocular vision and lack of stereopsis [33]. We found only one report that specifically explored the motor development of children with amblyopia that were not part of a more general group of strabismus patients [34]. In this report, children with amblyopia performed poorly on a motor proficiency test compared to their age-matched peers.

The objective of this paper was to explore further the postural stability of children with amblyopia, as well as those, who have strabismus without amblyopia. We elected to examine their postural control with an easy to use clinical test battery, the balance subtest of the Bruininks-Oseretsky Test of Motor Proficiency (BOT2) [35]. This widely accepted test can assess both the static and the dynamic balance functions [36–38]. It has been previously used to assess children with moderate to severe visual impairment and blindness [39, 40]. We compared amblyopic participants, who have both disrupted binocular vision and reduced visual acuity in one eye, to participants with strabismus without amblyopia, who have near equal vision in both eyes, but have some level of binocular dysfunction. We hypothesized that the amblyopia group and the strabismus without amblyopia group would both perform poorly compared to children with normal vision. We also predicted that the amblyopia group would perform slightly worse than the strabismus group, as this group has an extra visual deficit to contend with (the reduced and unequal visual acuity).

## Methods

The research study was approved by the Research Ethics Board at The Hospital for Sick Children, Toronto, Ontario, Canada, and conformed to the guidelines of the Declaration of Helsinki. Written consent and verbal assent were obtained from the guardians and the children, respectively.

### Participants

In this cross-sectional cohort study, participants aged 4–21 years were recruited from the Ophthalmology and Otolaryngology Clinics at The Hospital for Sick Children, Toronto. All underwent a standard eye examination that was performed by a certified orthoptist or a pediatric ophthalmologist for the following: visual acuity using the Early Treatment of Diabetes Retinopathy Study (ETDRS) test, refractive errors, Worth 4 Dot/ Bagolini test for sensory fusion, stereoacuity (Randot), and eye alignment (cover–uncover test and alternate prism cover test). Patients were excluded if they had: (1) any ocular pathology; (2) previous intraocular surgery; (3) presence of hearing impairment; (4) any specific neurological disease (e.g. stroke, multiple sclerosis, brain tumour, etc.); (5) any other neurodevelopmental or behavioural disorder (e.g. autism spectrum disorder); (6) restrictive or neurological basis for the strabismus, or (7) strabismus surgery within 6 months prior to the balance test. None of the participants complained of diplopia.

Children were divided into three groups: (1) normal vision (N = 22) defined by a visual acuity of at least 20/25 (0.1 logMAR) in both eyes; (2) strabismus without amblyopia (N = 16) with normal visual acuity in both eyes); and (3) unilateral amblyopia (N = 18) (strabismic amblyopia or mixed mechanism). Ambylopia was defined as: (1) best corrected visual acuity between 20/40 (0.3 logMAR) and 20/400 (1.3 logMAR) in the amblyopic eye; (2) visual acuity of 20/25 (0.1 logMAR) or better in the non-amblyopic eye; and (3) an inter-ocular acuity difference ≥0.2 logMAR. Strabismic amblyopia was defined as amblyopia: (1) in the presence of either a heterotropia at distance, near fixation or both, or a history of strabismus surgery; and (2) in the absence of refractive error meeting the criteria below for combined mechanism amblyopia. Combined mechanism was defined as amblyopia in the presence of: (1) either a heterotropia at distance or near fixation or both, or a history of strabismus surgery; and (2) anisometropia of ≥1.0D spherical equivalent or ≥1.5D of difference in astigmatism on any meridian, which persisted after 12 weeks of spectacle correction. Severity of amblyopia was defined according to PEDIG [41] as mild/moderate for visual acuity between 20/40–20/100 (0.3–0.7 logMAR) in the amblyopic eye and as severe for visual acuity between 20/120-20/400 (0.78–1.3 logMAR) in the amblyopic eye. The patients in the strabismus without amblyopia group had visual acuity of at least 20/25 (0.1 logMAR) in both eyes, in the presence of either a heterotopia at distance, at near fixation, or both, or a history of strabismus surgery (or botulinum injection). The normal vision group were siblings of patients that were recruited from the waiting area of both clinics. They were unrelated to participants in the study groups.

All participants passed a manual hearing screen test. The hearing test was performed using a standardized audiometer (Model MA27 Audiometer, Maico Diagnostics, Eden Prairie, MN 55344, USA) and standard TDH 39/41 headphones. To pass the screening test, the participants had to indicate detection of tones at 20–25 dB at least twice at each of the following frequencies: 500, 1000, 2000 and 4000Hz. This is in accordance with the (American National Standards Institute) ANSI 1969/ANSI 1971 recommendations [42]. Presentation of pure tones was randomized by frequency. Each participant was seated so that they could not see the examiner operating the audiometer and was asked to raise their hand when they heard a tone being presented.

### The BOT2 balance subtest

The Bruininks-Oseretsky Test of Motor Proficiency (BOT2) is a clinical test battery that was first introduced in 1978. It was designed to provide educators, clinicians, and researchers with a clinical assessment tool for motor proficiency [43]. It can be used to evaluate motor dysfunction and developmental delay. It is a standardized age-referenced test that is widely-used in Physical Therapy and Occupational Therapy. The test is comprised of several subtests to assess both gross and fine motor skills. The balance subtest consists of nine separate tasks, some of which are performed with both eyes open and eyes closed, and can assess both dynamic and static postural control. A detailed summary of the balance subtest procedures can be found in a previous publication [36]. In brief, BOT2 is comprised of seven static assignments, which are performed either standing on a line on the floor or on a small balance beam while visual fixation is held at distance of approximately 3 meters (Table 1). The children are asked to hold their position for at least 10 seconds. Each task is timed (in seconds) until the participant loses balance (defined as putting the raised foot on the ground–for example) within a maximum period of 10 seconds. There are also two dynamic tasks: (1) walking along a line; and (2) walking heel to toe (i.e. tandem walking) along a line. At least 6 steps warrant a full score. The raw score from each of the tasks is ranked and receives a point score (0–4 or 0–5 depending on the task). The point scores of the nine separate tasks are summed to produce a total point score (range: 0–37 points). The total point score is then compared to an age- and gender-matched scale score based on normative population data, provided as part of the test, and converted into a corrected point score (range, 1–35 points). Of the seven static balance control assignments, 3 were performed twice: once with eyes open (EO) and again with the eyes closed (EC). The tandem stand with heel to toe on the balance beam was performed only with eyes open.

The BOT2 was administered by a single examiner (ABZ). Each participant was given two chances to complete each of the task for all conditions and the best score achieved was counted toward the total performance score.

**Table 1:** Bruininks-Oseretsky test of motor proficiency- balance subtest static tasks.

Balance Subtest Tasks*	Eyes Open vs Closed	Task Number
Tandem stand with feet apart on the floor along a line	Open Closed	1 4
One-leg stand on the floor along a line	Open Closed	3 6
Tandem stand heel to toe on a beam	Open	7
One-leg stand on a beam	Open Closed	8 9

*The two dynamic tasks, task 2, walking along a line and task 5, walking heel to toe along a line were not included in this study.

### Statistical analysis

The omnibus statistical significance of the difference in final corrected BOT2 score between the three groups (amblyopia, strabismus without amblyopia and visually-normal controls) was determined using one-way analysis of variance (ANOVA). When the performance on each specific task was compared among the three groups, a linear regression model was used with gender and age as confounding factors. The statistical significance level was set at p-value less than 0.05 for all purposes. A two-way mixed repeated-measures ANOVA was utilized to assess the relative influence of eyes open (EO) vs eyes closed (EC) on the performance of the three participant groups on three different tasks. Chi-square analysis was used to compare categorical values between the groups, including gender distribution, previous surgery, and presence of stereopsis. All statistical analyses were performed using the SPSS version 22.0 (IBM Corporation and other(s) 1989, 2013, Armonk, North Castle, New York, United States).

## Results

### Clinical characteristics

Clinical data from the subjects in the amblyopic and the strabismus without amblyopia groups are presented in S1 and S2 Tables. There were 18 patients with amblyopia (8 males; mean age ±SD 8.5±2.0; age range 5.6–12.4 years), 16 patients with strabismus only without amblyopia (10 males; mean age ±SD 10.9±3.6; age range 6.5–17.8 years) and 22 children with normal vision (10 males; mean age ±SD 10.6 ± 3.2; age range 5.6–17.3 years). Tukey’s method for multiple comparisons showed that the groups were not significantly different by age [F (2, 55) = 2.55 p = 0.09]. The groups were also similar by gender distribution (Chi-Square value = 1.404 df = 2 p = 0.50). All but one of the amblyopic participants had mild/moderate amblyopia with a BCVA ranging from 0.3–0.7 logMAR. Of the amblyopic patients, twelve participants had strabismic amblyopia and six had mixed mechanism amblyopia (S1 Table).

In the strabismus only without amblyopia group (n = 16), 9 children had esotropia (or consecutive post-surgical exotropia) at the time of evaluation and 7 had exotropia, mainly intermittent exotropia XT). Previous surgery was documented in 8/16 strabismus patients, which was not statistically significant from the amblyopic patients, 12/18 of whom had surgery (Chi-Square value = 0.97 df = 1 p = 0.32). Some level of stereopsis was documented in ten patients with strabismus only (3000 seconds or better); eight of these patients had finer levels of stereo-acuity ranging from 20 to 60 seconds. This was significantly higher compared to the amblyopia group, where only three children had some level of stereopsis; two had 3000 seconds and one had 140 seconds (Fischer’s Exact Test p<0.0001).

### Balance in children with amblyopia and strabismus without amblyopia

**Fig 1:**
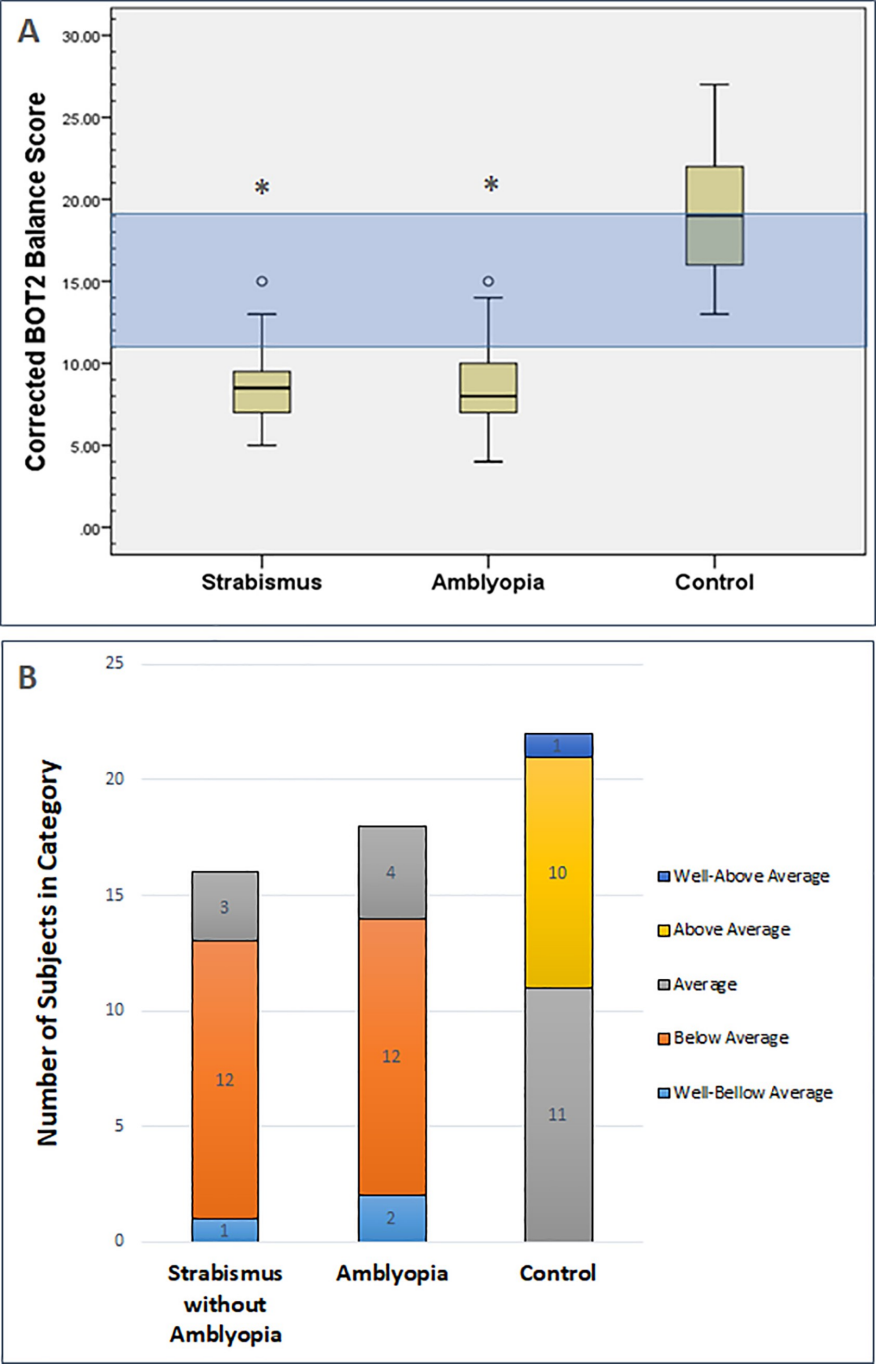
Age and gender adjusted BOT2 score among the different groups. Diagram A is a boxplot illustrating the distribution of the BOT2 corrected point score for age and gender (range 1–35) among the three groups: normal controls, strabismus only without amblyopia group and the amblyopia group. The definition of the corrected point score is included in the methods section. The inter-quartile area of the strabismus only group and the amblyopia are much lower (7.0–9.25 score and 7.0–10.25 score, respectfully) compared to the control group 15.75–22.25 score. The blue area in the graph is the average corrected score level (11–19) according to descriptive categories of the BOT2 test. The strabismus only group (mean score 8.6 ± 2.4 SD) and the amblyopia group (mean score 9.0 ± 3.1 SD) had much lower scores compared to the normal controls group (mean score 18.9 ± 4.2 SD) [F (2, 53) = 60.62, px10^-3^], but the mean corrected BOT2 score of these two groups was not statistically different. Diagram B shows the distribution of the BOT2 corrected scale score according to five descriptive categories of performance based on the BOT2 test’s nomogram (Well-Above Average, Above Average, Average, Below Average and Well-Below Average). The graph demonstrates the number of subjects distributed in each descriptive category among the three study groups (strabismus only group, amblyopia group and normal controls). The performance of all of the subjects in the control group was categorized as average and above, while most of the strabismus group and the amblyopia group performed below average. ^o^ Signifies an outlier that is outside of the 95 percentile. * Signifies statistically significant difference from normal controls using one-way ANOVA.

The age and gender-adjusted BOT2 balance scores were substantially reduced in the amblyopia group (mean score 9.0 ± 3.1 SD) and the strabismus without amblyopia group (mean score 8.6 ± 2.4 SD) compared to visually normal children (mean score 18.9 ± 4.2 SD) [F (2, 53) = 60.62, px10^-3^], but no statistical difference was demonstrated between the two patient groups (p = 0.907) (Data are displayed in S3 Table). The distribution of the corrected scores was further analyzed with boxplots (Fig 1A), showing that the inter-quartile range for both the strabismus only group and the amblyopia group (7.0–9.25 score and 7.0–10.25 score, respectfully) was not only lower than the control group 15.75–22.25 inter-quartile range, but also much lower than the average descriptive category (11–19) of the BOT2 test’s nomogram.

The BOT2 corrected scale score can be further described according to five descriptive categories based on the BOT2 test’s nomogram (Well-Above Average, Above Average, Average, Below Average and Well-below Average). Fig 1B demonstrates the distribution of the corrected balance score among the three groups according to these five categories. This figure highlights again that most of the subjects in the strabismus only group (13/16) and the amblyopia group (14/18) performed below average, while none of the controls were below average (See S4 Table for more details on the descriptive categories of the BOT2 test).

#### Effect of type of strabismus on balance

In the strabismus only group without amblyopia, the age and gender-adjusted BOT2 scores were not statistically different between the Esotropia group (mean±SD = 8.0±1.7) and Exotropia group (mean±SD = 9.9±3.1; t (14) = 1.51, p = 0.15]. The level of binocular function was different among the two groups with only one out of the nine esotropic patients (11%) possessing good stereopsis of 60 sec or better. By contrast, all of the exotropia patients had good stereopsis (Fisher’s Exact Test p = 0.001). Despite better binocular function in the exotropic group, performance was similar in both groups on the balance test.

#### Effect of binocular vision on balance

When comparing the age and gender-adjusted BOT2 score of the strabismus only patients with good stereopsis 60 sec or better (9.8±3.0) to patients with 3000 sec or no stereopsis (7.9±1.7), no statistical difference was found between the two groups [t(14) = -1.55, p = 0.144].

#### Effect of size of deviation on balance

Within the strabismus only group, the performance of patients with small angle strabismus 10 prism diopters (PD) or less and intermittent strabismus (mean angle of deviation 6.8±2.3 SD) was compared to patients with larger angles (mean angle of deviation 26.5±9.5 SD). The small angle group mean on the balance subtest of the BOT2 test was 9.0±3.5 compared to the large angle group of 8.7±2.2; this not statistically different [t (14) = -0.9, p = 0.85].

#### Performance on individual tasks within the BOT2 balance subtest

**Fig 2:**
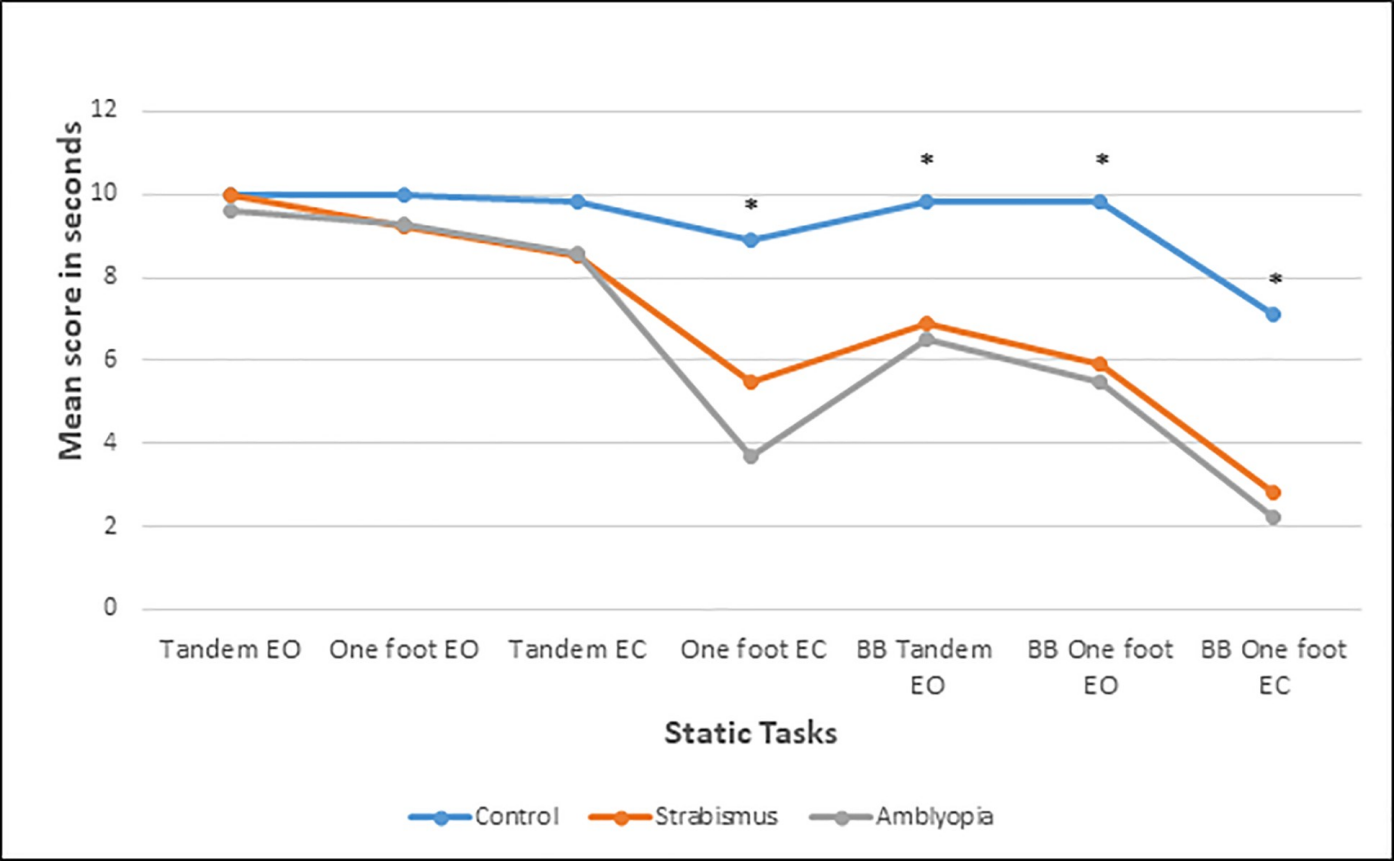
Means of individual static balance tasks scores among the groups. The diagram illustrates the difference in means of the individual task time in seconds (time to loss of balance) among the different groups. Included in this analysis are the static tasks: 1- Tandem stand with eyes open (EO), 3- One-foot stand EO, 4- Tandem with eyes closed (EC), 6- One-foot stand EC, 7- One-foot stand on a balance beam (BB) EO, 8- Tandem stand on a beam EO, 9- One-foot stand on a beam EC. Task 6–9 demonstrated a statistically significant difference between the two patient groups and the visually normal controls. Both groups were not statistically different from each other. * Signifies a statistically significant difference among the groups using one-way ANCOVA with age and gender as co-variant. EO = eyes open. EC = eyes closed. BB = balance beam.

When analyzing individual tasks within the balance assessment of the BOT2 test using a one-way ANCOVA (analysis of covariance), it became apparent that the amblyopia group and the strabismus without amblyopia group perform significantly poorer compared to controls on specific tasks (Fig 2). Since we had no age or gender matched tables to compare to on the individual tasks, we had to correct for age and gender using linear regression. The time until the participant lost balance in seconds was used for this specific analysis. We only included the static tasks in this analysis: 1- Tandem stand with eyes open (EO), 3- One-foot stand with eyes open (EO), 4- Tandem with eyes closed (EC), 6- One-foot stand with eyes closed (EC), 7- One-foot stand on a beam EO, 8- Tandem stand on a beam EO, 9- One-foot stand on a beam EC. For tasks 1, 3, and 4 no statistical difference was found between the three groups [Task 1, F (2, 51) = 0.64, p = 0.53; Task 3, F (2, 51) = 1.48, p = 0.24; Task 4, F (2, 51) = 2.75, p = 0.07]. Tasks 6, 7, 8 and 9 demonstrated a significant difference between the three groups [Task 6, F (2, 51) = 21.27, p<0.001; Task 7, F (2, 51) = 7.92, p = 0.001; Task 8, F (2, 51) = 14.39, p<0.001; Task 9, F (2, 51) = 29.21, p<0.001]. Post hoc tests showed a significant difference between the two patient groups and visually normal controls on all four tasks 6–9 (For tasks 6, 8, 9, p<0.001 for both strabismus only group and amblyopia group compared to controls; for task 7, p = 0.003 for strabismus only group compared to controls and p = 0.007 for amblyopia group compared to controls). However, no statistical significant difference was found between the amblyopia group and the strabismus only without amblyopia group (p = 1.0 for all tasks).

**Fig 3:**
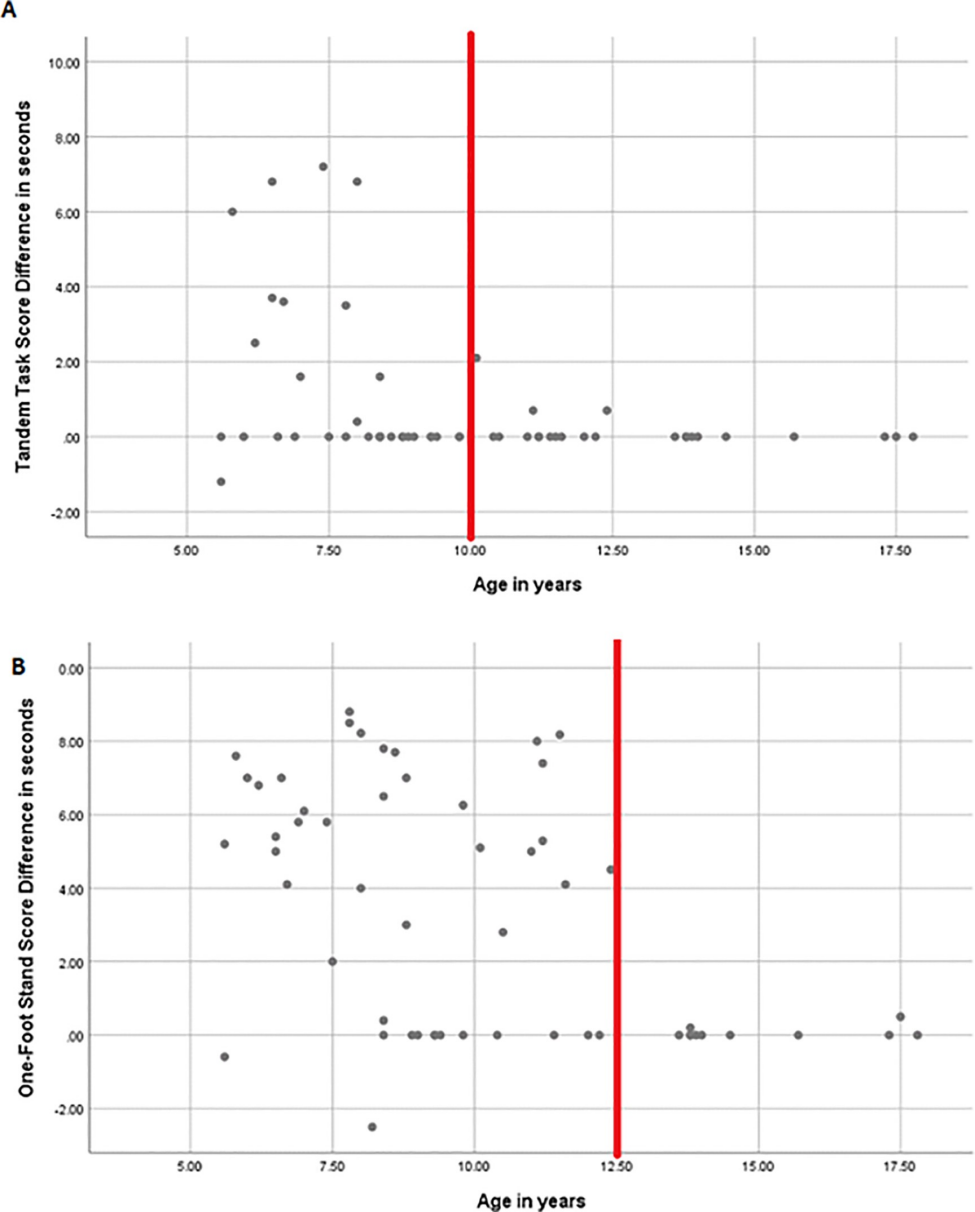
Age influences the difference in performance between viewing conditions. This scatter plot demonstrates the effect age has on the performance between viewing conditions (eye open vs eyes closed). The y-axis is the difference between the performance measured in seconds (time to loss of balance), while the eyes are open (EO), versus the time to loss of balance while the eyes are closed (EC). The BOT-2 scores have age-adjusted standards for the balance subtest, however the data presented in these graphs are not corrected for age, as this information is not available for specific tasks. Fig 3(A) shows that on the tandem task this difference in performance between viewing conditions is markedly reduced in patients older than 10 years of age. Fig 3(B) shows that on the one-foot stand this difference is reduced in participants older than 12.5 years of age. The red line signifies the approximate age, beyond which the difference between viewing conditions is markedly reduced and no longer variable. The older the kids are the difference in their performance is less influenced by the viewing conditions, and they perform with their eyes closed almost as well as with their eyes open.

**Fig 4:**
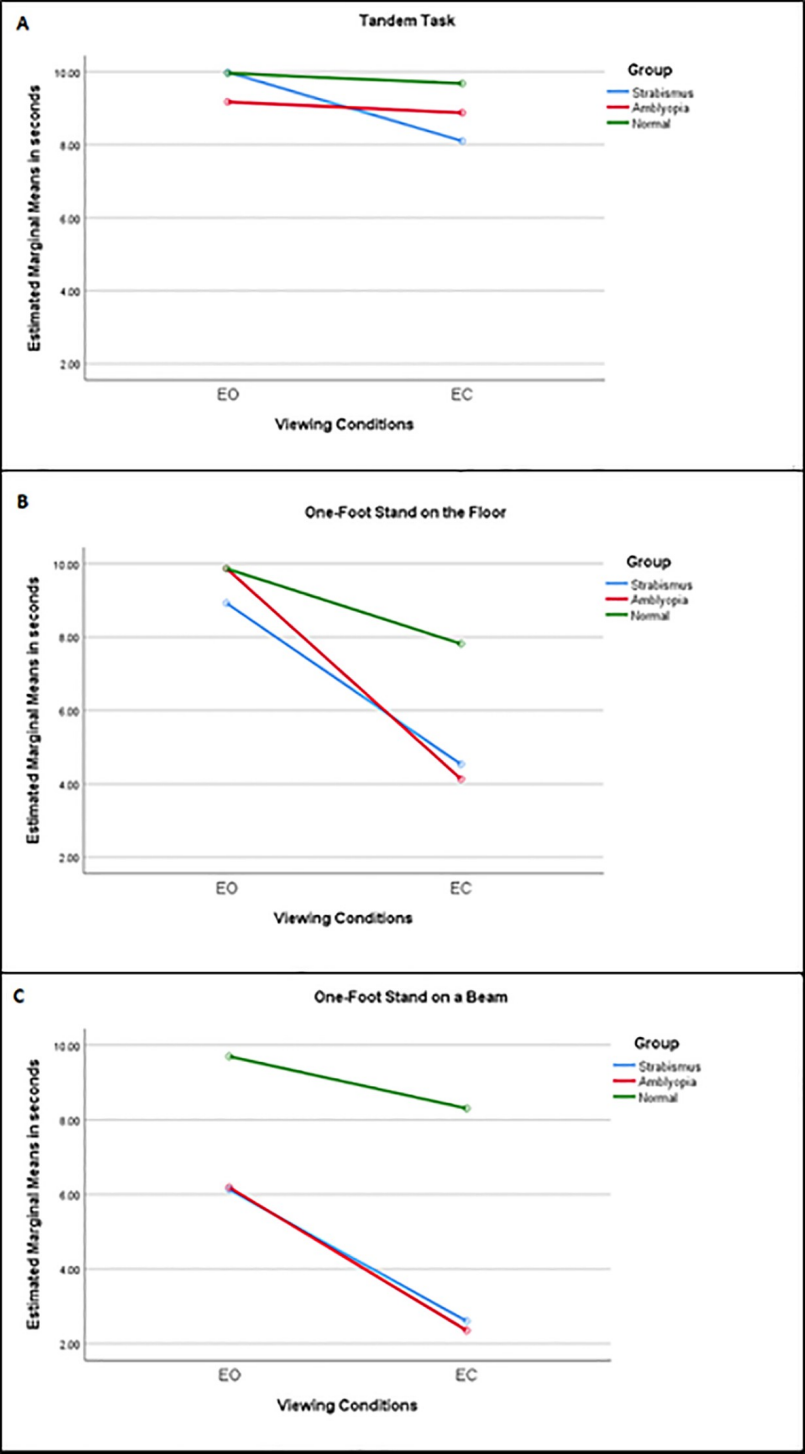
Mean score of three static tasks among the groups in different viewing conditions. The diagram illustrates the difference in means of three different tasks the tandem task (A), the one-foot stand task (B) and the one-foot stand on a beam (C). The performance was measured as time to loss of balance in seconds with the eyes open (EO) and with the eyes closed (EC) among the different groups. Using the one-way repeated measures ANCOVA the amblyopia and the strabismus only group performed worse with their eyes closed compared to normal controls on the tandem and the one-foot stand (p<0.001), but this was not statistically significant on the one-foot stand on a beam (p = 0.25). The results presented in this figure are for males. Similar reduction in performance while the eyes were closed were also demonstrated in females. *Covariates appearing in the model are evaluated with the age value at 10.03 years. EO = eyes open. EC = eyes closed.

The performance of participants in all groups was reduced once the patient’s eyes were closed. Hence, the interaction between the Viewing Condition [eyes open (EO) vs eyes closed (EC)] and Group type was tested on three different tasks: tandem, one-foot stand and one-foot stand on a beam. The performance of each of the groups, strabismus only without amblyopia, amblyopia and visually normal controls, was analysed using ANCOVA with repeated measures (within-subjects factor was Viewing Condition, EO vs EC; between-subjects factors included both Group type and Gender with the co-variant Age). Again, the longer the time until the participant lost balance in seconds was used for the analysis. The participants’ performance was significantly reduced when the eyes were closed on the tandem task [F (1, 49) = 15.59, p<0.001] and the one-foot stand [F (1, 49) = 36.60, p<0.001] (Tables 2 and 3). However, the reduction in performance on the one-foot stand on a beam was not significant [F (1, 49) = 1.37, p = 0.25], because the performance was reduced even with the eyes open (Table 4). Age also influenced the subjects’ performance on all tasks [Tandem task, F (1, 49) = 9.12, p = 0.004; one-foot stand, F (1, 49) = 29.02, p<0.001; one-foot stand on a beam, F (1, 49) = 4.82, p = 0.03] with greater difference in balance performance between EO vs EC in the younger age. This difference in performance between viewing conditions was not noticeable in patients older than 10 years of age on the tandem task and older than 12.5 years of age on the one-foot stand (Fig 3). This clear cut-off was not as prominent on the one-foot stand on a beam task, but beyond 10 years of age, this difference between viewing conditions was reduced. No interaction was found between Viewing conditions and Group type on the tandem task [F (2, 49) = 1.84, p = 0.17]. However, significant interactions between the Viewing conditions and Group type were found on both the one-foot stand task [F (2, 49) = 29.02, p<0.001] and the one-foot stand on a beam task [F (2, 49) = 30.27, p<0.001] (Fig 4). On the pairwise comparisons, both the amblyopia group and the strabismus only group performed significantly worse compared to controls (p<0.001 in both tasks the one-foot stand and the one-foot stand on the beam), but the clinical groups were not statistically different from each other (p = 1.0 on both tasks).

**Table 2:** Balance score analysis on the tandem task using ANCOVA with repeated measures.

Source	df	F	P Value
Viewing Conditions (eyes closed vs eyes open)	1, 49	15.591	.000^a^
Viewing Conditions * Age	1, 49	9.470	.003^a^
Viewing Conditions * Group	2, 49	2.607	.084
Viewing Conditions * Gender	1, 49	.026	.873
Viewing Conditions * Group * Gender	2, 49	.435	.650
Age	1, 49	9.115	.004^a^
Group	2, 49	1.844	.169
Gender	1, 49	.603	.441
Group * Gender	2, 49	.261	.771

The model used for this analysis was ANCOVA (analysis of covariance) with repeated measures. The dependent within-subjects variable was Viewing Conditions (eyes opens vs eyes closed), whereas the between-subjects factors were Group type and Gender with the co-variant Age.

The asterisk signifies the interaction between variables in the regression model.

^a^ Significance at P<0.05.

**Table 3:** Balance score analysis on the one-foot stand task using ANCOVA with repeated measures.

Source	df	F	P Value
Viewing Conditions (eyes closed vs eyes open)	1	36.604	.000^a^
Viewing Conditions * Age	1	11.474	.001^a^
Viewing Conditions * Group	2	10.129	.000^a^
Viewing Conditions * Gender	1	2.855	.097
Viewing Conditions * Group * Gender	2	.046	.955
Age	1	29.016	.000^a^
Group	2	17.247	.000^a^
Gender	1	2.391	.128
Group * Gender	2	.528	.593

The model used for this analysis was ANCOVA with repeated measures. The dependent within-subjects variable was Viewing Conditions (eyes opens vs eyes closed), whereas the between-subjects factors were Group type and Gender with the co-variant Age.

The asterisk signifies the interaction between variables in the regression model.

^a^ Significance at P<0.05.

**Table 4:** Balance score analysis on the one-foot stand on a beam task using ANCOVA with repeated measures.

Source	df	F	P Value
Viewing Conditions (eyes closed vs eyes open)	1, 49	1.367	.248
Viewing Conditions * Age	1, 49	1.522	.223
Viewing Conditions * Group	2, 49	1.973	.150
Viewing Conditions * Gender	1, 49	3.046	.087
Viewing Conditions * Group * Gender	2, 49	.205	.816
Age	1	4.816	.033^a^
Group	2	30.266	.000^a^
Gender	1	.060	.808
Group * Gender	2	2.043	.141

The model used for this analysis was ANCOVA with repeated measures. The dependent within-subjects variable was Viewing Conditions (eyes opens vs eyes closed), whereas the between-subjects factors were Group type and Gender with the co-variant Age.

The asterisk signifies the interaction between variables in the regression model.

^a^ Significance at P<0.05.

## Discussion

Our study demonstrated reduced balance control in children with both amblyopia and strabismus only without amblyopia. The strabismus without amblyopia group in this cohort did not show better balance control compared to the amblyopic patients, despite this group’s better visual acuity and better binocular function. Both groups performed as poorly on our clinical test battery compared to the visually normal controls. All of our amblyopic patients but one had mild-moderate amblyopia. It is possible that we could have found a difference between the groups if the amblyopia group had included participants with more severe amblyopia. It is also possible that some fine developmental deficit (fine balance impairment e.g., otolith dysfunction) exists among strabismus patients whether they are amblyopic or not.

Our present results measured using the BOT-2 test are consistent with previous findings of impaired postural control measured in individuals with strabismus using a posturography platform in a research laboratory setting [23, 25, 27, 31]. In addition, parents of children with strabismus and amblyopia report significantly more balance deficits than parents of children with normal vision on a sensorimotor development questionnaire (the Infant Developmental Skills Survey), [29, 34]. The advantage of the BOT2 balance test is that it is applicable to the clinic; it can be easily administered in an office examination, does not require special equipment, is widely accepted and has been in use for many years [38] in both clinical and research environments [36].

Earlier studies on strabismus and balance assessed patients with various strabismus types. Postural abnormalities were recorded in both esotropic [23, 25, 30] and exotropic patients [31]. Some studies grouped patients with esotropia, exotropia, and vertical strabismus into one group [24, 26, 31]. One of the pitfalls of our study was that our group of strabismus only patients was also diverse. Hence, we compared the performance of the esotropic patients within the strabismus only group to patients with exotropia (including intermittent exotropia). We were not able to demonstrate a statistically significant difference between the two patients groups. This is contradictory to previous reports [23, 31, 44, 45], which demonstrated better postural stability in esotropic children compared to children with divergent strabismus.

All patients with exotropia in the strabismus only group in our cohort had some form of intermittence or phoria, and therefore achieved better binocular function with stereopsis of 60 arc seconds or better, compared to the esotropic patients, who had constant tropia and worse stereopsis. So, comparing esotropia to exotropia groupings, in our cohort, was in essence also demonstrating that there was no difference in performance between tropia versus phoria/intermittent strabismus To further emphasize this point, we specifically compared patients with good stereopsis to patients with poor stereopsis, and could not demonstrate a statistically significant difference between the two groups on the balance subtest of the BOT2 [t (14) = -1.55, p = 0.144]. This is contradictory to one previous report that demonstrated a reduction in balance with reduced stereoacuity [31]. Our study implies that abnormal vergence control may be driving the decreased static postural control in children with strabismus and amblyopia. Children with normal vestibular and somatosensory function may have difficulties maintaining balance due to vergence dysfunction and latent strabismus despite some binocular function [21, 22]. In several studies, reduced balance control was not only demonstrated in children with heterotropias, but it was also exhibited in children with milder binocular dysfunction, including children with reduced vergence amplitudes and intermittent strabismus with good stereopsis [21, 22].

It has been previously implied that correcting strabismus can improve the motor development of children with strabismus [25, 27, 29, 30], but there are some contradicting reports [31]. A few reports demonstrate improved postural control 2–3 months post-operatively [25, 27, 30]. However, Matsuo and coworkers tested their patient’s immediately after surgery (three days post-op) and found their stability was worse post-op [31]. This is probably due to the need to adapt (motor-wise and sensory-wise) to the new alignment. Proprioceptive information from the extra-ocular muscles is altered by strabismus surgery [46], particularly in the first two months after surgery [47]. Hence, in our study we excluded patients that had surgery within 6 months prior to the balance test. It is possible that correcting certain types of strabismus early during a critical period may improve motor development and balance control. This highlights a potential for future research to address whether balance deficits occurring in development in patients with strabismus and amblyopia might be reversible with restoration of binocular vision. The presence of a sensitive period for establishing balance should be further explored.

Some of the balancing tasks on the BOT-2 clinical test battery were performed with different viewing conditions, with the eyes open and as well as with eyes closed. This enabled us to explore the role of vision in the balance of children with amblyopia and strabismus. The relative contribution of the visual system to balance control is greater in children relative to adults [7, 8, 14]. In our study, balance was poorer for all groups on several tasks, when their eyes were closed compared to binocular viewing. This is consistent with previous findings documenting a twofold decrease in postural stability in individuals when their eyes are closed [48–50]. The one foot stand on the floor with the eyes closed stood out as a simple and powerful tool to screen for balance impairment. It demonstrated the greatest difference between the groups without the need for special equipment, making it a good candidate for a simple screening tool as part of a more comprehensive clinical assessment.

Significant interactions between the Viewing conditions and Group type were revealed by more challenging tasks—the one foot stand on the floor and on the one foot stand on a balance beam. Viewing conditions (eyes closed vs eyes open) affected the performance of the participants differently in each group. We hypothesized that the visual contribution to balance control would be lower in children with visual impairment. We expected that children with amblyopia and strabismus without amblyopia would rely more on the input from their vestibular and somatosensory systems for postural control compared to visually normal controls. In support of this hypothesis, Dickmann et al [24] showed that individuals with strabismus perform better than visually normal controls in the dark with their eyes closed. Yet, in our study both the amblyopia group and the strabismus without amblyopia group were more sensitive to eye closure and lost balance more easily, when their eyes were closed, compared to children with normal vision (p<0.001). This means that these children were not making good use of compensatory postural mechanisms and/or other sensory systems, including proprioception and vestibular input, to adapt to challenging balancing tasks.

Age also influenced our subjects’ performance on several tasks. The younger the participants were, the greater the difference in balance performance was noted between viewing conditions (eyes closed vs eyes open) on these assignments. In our cohort, this variability was most prominent in patients younger than 10 years of age, although it may continue past this age with more demanding tasks. This concurs with our current understanding of the influence of vision on balance and postural control [8, 51–53]. Balance control is established early on in life. It is dependent on the intricate interrelationship of three systems: the visual system, the vestibular and the somatosensory proprioceptive system. Postural stability improves as each system matures and the conflicts among the different sensory inputs are better resolved [8, 51–53]. This process of maturation occurs at a different pace for each sensory input. The proprioceptive system seems to dominate balance control at a very early stage in life, with some reports showing it reaches adult level of function as early as 3–4 years of age [8, 51]. In this sequential process of development, the visual contribution to balance control seems to develop next [8, 51, 52], reaching full maturation at about age 15 years. However, children at the age of 2–5 years seem to clearly rely on their vision for postural stability with the visual input dominating over other sensory inputs (proprioception and vestibular system) [7, 8, 13, 14]. This process continues until all of the systems are fully calibrated to adult level of performance, when the vestibular system seem to dominate [8, 51, 52]. Since balance is so dependent on visual inputs at such early stages of life, it is highly likely that disruption of the visual input due to impaired binocularity, even as fine as reduced vergence amplitudes can offset balance control, which is depicted in our present study of amblyopic patients and strabismus only without amblyopia patients. Yet, it is possible that our patients’ postural control may improve with time to adults’ level of stability, and that it just takes longer for them to mature to an adult’s level of function.

This study’s limitations include its diverse group of patients. The BOT2 balance subtest may not have been sensitive enough to pick up fine differences between the groups, hence the lack of difference between our two patients’ groups. However, one of its advantages was that the observer, performing the BOT2 clinical test, was masked to the amblyopia or strabismus group when running the testing.

## Conclusion

Our findings suggest that binocular vision plays an important role in the development and maintenance of balance control in children. Balance was reduced in both amblyopic patients and strabismus patients without amblyopia patients equally. It seems that even milder cases of intermittent strabismus and heterophorias, if present early on in life, can lead to some postural instability, despite higher levels of binocular function. Further study into the contribution of the various components of vision to balance control is warranted. Future research should also explore whether there is a sensitive period for establishing balance during development and whether balance deficits might be reversible with restoration of binocular vision.

## Supporting information

S1 Table Clinical characteristics of subjects in the amblyopia group. (DOCX)

S2 Table Clinical characteristics of subjects in the strabismus without amblyopia group. (DOCX)

S3 Table BOT2 balance scores among study groups. (DOCX)

S4 Table Descriptive categories of the BOT2 test. (DOCX)
