# Supplementary material for: Postural stability and visual impairment: Assessing balance in children with strabismus and amblyopia
Source: PLoS One. 2018 Oct 18;13(10):e0205857. doi: 10.1371/journal.pone.0205857 (PMC6193669; doi:10.1371/journal.pone.0205857)
Supplement: S1 Table — (DOCX) [file pone.0205857.s001.docx]

**S1 Table: Clinical Characteristics of Subjects in the Amblyopia Group**

| No. | Age (yrs) | Gender | Amblyopia Type | BCVA (logMar) | | Refraction | | Angle of Strabismus | | Stereo  (sec) | Surgery  (y/n) |
| --- | --- | --- | --- | --- | --- | --- | --- | --- | --- | --- | --- |
|  |  |  |  | RE | LE | RE | LE | Near | Dist |  |  |
| 1 | 11.2 | F | Strab | 0 | 0.3 | +2.00 | +1.75 | ET 25 | ET+E 12 | 3000 | Y |
| 7 | 7.4 | F | Strab | 0.4 | 0.1 | (+7.00+0.25x178) add +2.2 | (+7.25+0.25x90) add +2.25 | ET+E 16 | ET 8 | 3000 | N |
| 9 | 6.2 | F | Strab | 0.0 | 0.4 | 0 | 0 | ET 10 | ET 14 | 0 | Y |
| 10 | 8.0 | M | Strab | 0.0 | 0.3 | 0 | 0 | ET 35 | ET 30 | 0 | Y |
| 11 | 8.4 | M | Strab | 0 | 0.3 | +8.75+1.50x120 | +9.00+2.25x90 | ET 40 | ET 25 | 0 | N |
| 14 | 8.8 | F | Strab | 0.0 | 0.3 | +3.50 | +3.25+0.75x10 | LXT 4 LHT 3 | LXT 10 LHT 2 | 0 | Y |
| 15 | 7.0 | F | Mixed | 0.0 | 1.0 | +1.00 | +2.75+2.50x134 | XT 25 | LXT 20 | 0 | N |
| 16 | 8.4 | M | Strab | 0.3 | 0.0 | 0 | 0 | ET 16 | ET+ E 20 | 0 | Y |
| 18 | 7.8 | M | Mixed | 1.0 | 0.0 | +6.25+1.50x90 | +5.5+0.25x16 | ET 6 | ET 6 | 0 | N |
| 19 | 5.8 | M | Strab | 0.3 | 0 | 0 | 0 | X(T) 40 | XT 35 | 0 | N |
| 22 | 11.2 | F | Mixed | 0.0 | 0.3 | +1.00 | +5.00 | X 8 | LXT 14 | 140 | Y |
| 24 | 8.4 | F | Strab | 0.3 | 0.1 | -2.00+2.50x106 | -1.75+1.75x74 | XT+X 16 | XT 14 | 0 | Y |
| 25 | 9.8 | M | Mixed | 1.0 | 0.0 | +2.75 | +0.75 | ET 14 | ET 10 | 0 | N |
| 29 | 6.9 | F | Mixed | 0.0 | 0.4 | Plano | -9.50+2.25x110 | X 10 | LXT 18 LHT 8-10 | 0 | Y |
| 31 | 11.5 | M | Mixed | 0.3 | 0.0 | +3.00+1.75x100 | +2.00+1.50x90 | X(T) 12 | X(T) 12 | 0 | Y |
| 32 | 7.4 | M | Strab | 0.6 | 0.1 | +6.00 | +5.00 | RET+E 6 | ET+E 4 | 3000 | N |
| 33 | 6.2 | F | Strab | 0.1 | 0.8 | +3.25+1.25x90 | +3.25+1.25x90 | LET 40 | LET 30 | 0 | N |
| 34 | 8.0 | M | Strab | 0.7 | 0.1 | +5.25+0.25x95 | +6.25 | XT 10 | XT 12 | 0 | Y |
