# Supplementary material for: Postural stability and visual impairment: Assessing balance in children with strabismus and amblyopia
Source: PLoS One. 2018 Oct 18;13(10):e0205857. doi: 10.1371/journal.pone.0205857 (PMC6193669; doi:10.1371/journal.pone.0205857)
Supplement: S2 Table — (DOCX) [file pone.0205857.s002.docx]

**S2 Table: Clinical Characteristics of Subjects in the Strabismus without Amblyopia Group**

| No. | Age  (yrs) | Gender | BCVA  (logMar) | | Refraction | | Angle of Strabismus (PD) | | Stereo  (sec) | Surgery  (y/n) | Amblyopia Treatment |
| --- | --- | --- | --- | --- | --- | --- | --- | --- | --- | --- | --- |
|  |  |  | RE | LE | RE | LE | Near | Dist |  |  |  |
| 2 | 6.5 | M | 0 | 0.1 | 0 | 0 | X 6 | X(T) 25 | 40 | N | N |
| 3 | 10.5 | F | 0 | 0 | +2.00 | +2.00 | ET+E 10 | ET+E 6 | 3000 | Y | N |
| 4 | 6.5 | F | 0.1 | 0.1 | +2.50 | +2.50 | ET 2 | ET 14 | 0 | Y | Y |
| 5 | 17.5 | M | 0 | 0 | 0 | 0 | XT 30 RHypoT | XT 30 RHypoT | 0 | Y | Y |
| 6 | 8.4 | M | 0.1 | 0 | +4.50 | +3.25 | ET 18 | ET 10 | 0 | Y | Y |
| 8 | 8.8 | M | 0.1 | 0.1 | +2.00 | +2.00 | ET14 | ET 18 | 0 | Y | N |
| 12 | 7.8 | M | 0 | 0 | 0 | 0 | LX(T) 10 | LXT 10 | 63 | Y | N |
| 13 | 11.6 | F | 0 | -0.1 | 0 | 0 | E 2 | ET4 | 40 | Y | N |
| 17 | 8.6 | M | 0 | 0 | -1.00 | -1.00 | XT 30 | XT 30 | 40 | N | N |
| 20 | 11.1 | M | 0.1 | 0 | 0 | 0 | XT 18 | XT 12 RHT 5 DVD | 0 | Y | Y |
| 21 | 10.1 | F | 0.1 | 0.1 | -0.75+1.50x108 | -0.50+1.50x97 | X(T) 53 | X(T) 30 LH(T) 5 | 20 | N | Y |
| 23 | 13.9 | F | -0.1 | -0.1 | 0 | 0 | X(T) 16 | X(T) 16 | 40 | Y | N |
| 26 | 13.8 | M | 0 | 0.1 | +4.75 | +4.75 | ET 2 | E(T) 6 L>R DVD | 0 | Y | N |
| 27 | 13.8 | M | 0.1 | 0 | +4.00 | +4.00 | ET+E 10 | ET+E 8 | 3000 | Y | N |
| 28 | 8.0 | M | 0 | 0.1 | Plano | Plano | X 8 | XT 20 LHT 2 | 40 | N | N |
| 30 | 17.8 | F | -0.1 | 0 | +1.75 | +1.75 | E(T) 10 | E 2 | 40 | N | N |
