# Supplementary material for: Postural stability and visual impairment: Assessing balance in children with strabismus and amblyopia
Source: PLoS One. 2018 Oct 18;13(10):e0205857. doi: 10.1371/journal.pone.0205857 (PMC6193669; doi:10.1371/journal.pone.0205857)
Supplement: S3 Table — (DOCX) [file pone.0205857.s003.docx]

**S3 Table: BOT2 Balance Scores among Study Groups**

| No. | Group | Corrected Balance Scale Score | Task 1-  Time to loss of balance (sec) | Task 3-  Time to loss of balance (sec) | Task 4-  Time to loss of balance (sec) | Task 6-  Time to loss of balance (sec) | Task 7-  Time to loss of balance (sec) | Task 8-  Time to loss of balance (sec) | Task 9-  Time to loss of balance (sec) |
| --- | --- | --- | --- | --- | --- | --- | --- | --- | --- |
| 1 | Amblyopia | 7 | 10 | 10 | 10 | 2.6 | 10 | 4.6 | 3.2 |
| 2 | Strabismus | 7 | 10 | 8.4 | 3.2 | 3.0 | 10 | 4.3 | 3.2 |
| 3 | Strabismus | 9 | 10 | 10 | 10 | 7.2 | 8.8 | 10 | 2.9 |
| 4 | Strabismus | 8 | 10 | 7.5 | 6.3 | 2.5 | 4.5 | 4.3 | 3.0 |
| 5 | Strabismus | 9 | 10 | 10 | 10 | 9.5 | 10 | 3.5 | 1.5 |
| 6 | Strabismus | 5 | 10 | 2.8 | 10 | 2.4 | 3.8 | 1.9 | 2.5 |
| 7 | Amblyopia | 6 | 10 | 8.5 | 10 | 5.5 | 1.5 | 1.5 | 0.9 |
| 8 | Strabismus | 7 | 10 | 10 | 10 | 2.2 | 3.8 | 4.0 | 2.2 |
| 9 | Amblyopia | 7 | 10 | 10 | 6.5 | 1.2 | 5.8 | 1.2 | 2.2 |
| 10 | Amblyopia | 11 | 10 | 10 | 10 | 5.9 | 10 | 10 | 4.0 |
| 11 | Amblyopia | 8 | 10 | 10 | 10 | 2.3 | 1.2 | 10 | 4.3 |
| 12 | Strabismus | 8 | 10 | 10 | 4.0 | 2.4 | 0.5 | 4.1 | 1.5 |
| 13 | Strabismus | 7 | 10 | 10 | 9.3 | 2.0 | 10 | 6.6 | 2.5 |
| 14 | Amblyopia | 15 | 10 | 10 | 7.9 | 4.9 | 10 | 3.9 | 4.1 |
| 15 | Amblyopia | 13 | 10 | 10 | 10 | 10 | 10 | 10 | 2.6 |
| 16 | Amblyopia | 10 | 10 | 10 | 10 | 10 | 8.5 | 6.5 | 4.4 |
| 17 | Strabismus | 9 | 10 | 10 | 10 | 9.8 | 8.4 | 0.8 | 2.0 |
| 18 | Amblyopia | 8 | 10 | 10 | 3.2 | 1.8 | 3.2 | 1.6 | 3.0 |
| 19 | Amblyopia | 9 | 10 | 10 | 10 | 10 | 4.3 | 7.1 | 2.5 |
| 20 | Strabismus | 4 | 10 | 10 | 9.3 | 5.5 | 4.9 | 10 | 2.2 |
| 21 | Strabismus | 5 | 10 | 2.5 | 10 | 5.0 | 5.9 | 2.5 | 2.2 |
| 22 | Amblyopia | 7 | 10 | 6.8 | 4.0 | 1.6 | 1.4 | 4.7 | 1.6 |
| 23 | Strabismus | 8 | 10 | 9.3 | 2.8 | 3.5 | 3.0 | 10 | 5.4 |
| 24 | Amblyopia | 14 | 10 | 10 | 7.5 | 3.2 | 6.8 | 10 | 3.0 |
| 25 | Amblyopia | 9 | 2.8 | 10 | 9.6 | 6.0 | 7.1 | 10 | 1.8 |
| 26 | Strabismus | 11 | 10 | 10 | 10 | 3.0 | 10 | 10 | 2.3 |
| 27 | Strabismus | 8 | 10 | 9.1 | 8.4 | 3.0 | 3.5 | 1.6 | 1.2 |
| 28 | Strabismus | 15 | 10 | 10 | 10 | 10 | 10 | 1.2 | 1.2 |
| 29 | Amblyopia | 7 | 10 | 10 | 10 | 1.5 | 2.0 | 10 | 0.8 |
| 30 | Strabismus | 9 | 10 | 10 | 10 | 4.7 | 8.7 | 0.9 | 0.4 |
| 31 | Amblyopia | 10 | 10 | 10 | 8.4 | 3.5 | 10 | 10 | 3.8 |
| 32 | Amblyopia | 10 | 10 | 10 | 10 | 3.7 | 10 | 10 | 3.4 |
| 33 | Amblyopia | 12 | 10 | 10 | 10 | 4.2 | 10 | 10 | 1.3 |
| 34 | Amblyopia | 7 | 10 | 10 | 10 | 1.8 | 10 | 10 | 1.8 |
|  | Control | 27 | 10 | 10 | 10 | 3.0 | 10 | 10 | 10 |
|  | Control | 17 | 10 | 10 | 10 | 3.0 | 10 | 7.0 | 4.29 |
|  | Control | 18 | 10 | 10 | 10 | 8.0 | 10 | 10 | 7.0 |
|  | Control | 20 | 10 | 10 | 10 | 10 | 10 | 10 | 9.0 |
|  | Control | 17 | 10 | 10 | 10 | 10 | 10 | 10 | 7.9 |
|  | Control | 20 | 10 | 10 | 10 | 10 | 10 | 10 | 10 |
|  | Control | 16 | 10 | 10 | 10 | 10 | 10 | 10 | 3.9 |
|  | Control | 24 | 10 | 10 | 10 | 10 | 10 | 10 | 10 |
|  | Control | 24 | 10 | 10 | 10 | 10 | 10 | 10 | 10 |
|  | Control | 13 | 10 | 10 | 10 | 10 | 10 | 10 | 2.9 |
|  | Control | 23 | 10 | 10 | 10 | 10 | 10 | 10 | 10 |
|  | Control | 23 | 10 | 10 | 10 | 10 | 10 | 10 | 10 |
|  | Control | 17 | 10 | 10 | 10 | 10 | 10 | 10 | 5.2 |
|  | Control | 22 | 10 | 10 | 10 | 10 | 10 | 10 | 10 |
|  | Control | 20 | 10 | 10 | 10 | 10 | 10 | 10 | 3.8 |
|  | Control | 15 | 10 | 10 | 10 | 10 | 10 | 10 | 4.6 |
|  | Control | 13 | 10 | 10 | 10 | 10 | 10 | 10 | 2.7 |
|  | Control | 13 | 10 | 10 | 10 | 5.0 | 10 | 10 | 7.3 |
|  | Control | 22 | 10 | 10 | 10 | 10 | 10 | 10 | 10 |
|  | Control | 22 | 10 | 10 | 10 | 10 | 10 | 10 | 10 |
|  | Control | 17 | 10 | 9.4 | 10 | 10 | 6.5 | 9.3 | 1.6 |
|  | Control | 13 | 10 | 10 | 6.4 | 5.9 | 10 | 10 | 1.3 |
