# Supplementary material for: Postural stability and visual impairment: Assessing balance in children with strabismus and amblyopia
Source: PLoS One. 2018 Oct 18;13(10):e0205857. doi: 10.1371/journal.pone.0205857 (PMC6193669; doi:10.1371/journal.pone.0205857)
Supplement: S4 Table — (DOCX) [file pone.0205857.s004.docx]

**S4 Table: Descriptive Categories of the BOT2 Test**

| Descriptive Category | Corrected Scale Score | Percentile Rank Range |
| --- | --- | --- |
| Well-above average | ≥25 | ≥98 |
| Above average | 20-24 | 84-97 |
| Average | 11-19 | 18-83 |
| Below Average | 6-10 | 3-17 |
| Well-below average | ≤5 | ≤2 |
